# Dietary Antioxidants in the Treatment of Male Infertility: Counteracting Oxidative Stress

**DOI:** 10.3390/biology10030241

**Published:** 2021-03-20

**Authors:** Elizabeth Torres-Arce, Barbara Vizmanos, Nancy Babio, Fabiola Márquez-Sandoval, Albert Salas-Huetos

**Affiliations:** 1 Center of Health Sciences, Institute of Translational Nutrigenetics and Nutrigenomics, Universidad de Guadalajara, 44340 Guadalajara, Mexico; jesseta@live.com (E.T.-A.); barbara.vizmanos@academicos.udg.mx (B.V.); 2 Andrology and IVF Laboratory, Division of Urology, Department of Surgery, University of Utah School of Medicine, Salt Lake City, UT 84108, USA; 3 Human Nutrition Unit, Biochemistry and Biotechnology Department, Universitat Rovira i Virgili, 43201 Reus, Spain; nancy.babio@urv.cat; 4 Institut d’Investigació Sanitària Pere i Virgili, 43204 Reus, Spain; 5 Consorcio CIBER, M.P., Fisiopatología de la Obesidad y Nutrición (ciBeRobn), Instituto de Salud Carlos III (ISCIII), 28029 Madrid, Spain

**Keywords:** reactive oxygen species, DNA fragmentation, male fertility, semen quality, antioxidants, foods, nutrients, supplements, dietary patterns, antioxidant paradox

## Abstract

**Simple Summary:**

The present review is a comprehensive description of reactive oxygen species (ROS’s) different sources, the re-productive consequences of excessive ROS and oxidative stress, and the possible treatments of ROS imbalances through antioxidant intake, foods, and dietary patterns to im-prove male infertility. In summary here we describe that some antioxidants, especially selenium and zinc, ω-3 fatty acids, CoQ10 and carnitines, have been positively related to sperm quality and therefore can help improving male sperm quality and fertility. However, excessive use of antioxidants may be detrimental to the spermatic function and many of the over-the-counter supplements are not scientifically proven to improve fertility. A long term and innocuous solution could be a balanced diet, as it takes advantage of the synergy of multiple antioxidants.

**Abstract:**

Infertility affects about 15% of the population and male factors only are responsible for ~25–30% of cases of infertility. Currently, the etiology of suboptimal semen quality is poorly understood, and many environmental and genetic factors, including oxidative stress, have been implicated. Oxidative stress is an imbalance between the production of free radicals, or reactive oxygen species (ROS), and the capacity of the body to counteract their harmful effects through neutralization by antioxidants. The purpose of this review, by employing the joint expertise of international researchers specialized in nutrition and male fertility areas, is to update the knowledge about the reproductive consequences of excessive ROS concentrations and oxidative stress on the semen quality and Assisted Reproduction Techniques (ART) clinical outcomes, to discuss the role of antioxidants in fertility outcomes, and finally to discuss why foods and dietary patterns are more innocuous long term solution for ameliorating oxidative stress and therefore semen quality results and ART fertility outcomes. Since this is a narrative review and not a systematic/meta-analysis, the summarized information in the present study should be considered cautiously.

## 1. Introduction

Infertility affects about 15% of the population and is defined as the inability to achieve a pregnancy after one year or more of sexual unprotected intercourse [1]. Male factors only, including decreased semen quality, are responsible for ~25-30% of cases of infertility [2]. Currently, the etiology of suboptimal semen quality is poorly understood, and many environmental and genetic factors, including oxidative stress, have been implicated [3]. Oxidative stress is essentially defined as an imbalance between the production of free radicals -also called reactive oxygen species (ROS)- and the capacity of the body to counteract their harmful effects through neutralization by antioxidants [4]. At normal physiological levels, ROS are essential to regulate many processes in reproduction, including sperm maturation and hyperactivation, acrosome reaction, or fertilization, among others; however, when ROS concentrations are too high many cellular processes are damaged [5,6]. Accumulating evidence from human and animal studies indicate that antioxidants and some components of the diet may play a pivotal role in modulating spermatogenesis by reducing the ROS presence in spermatozoa and semen plasma, and resetting the normal physiological levels [7]. The ROS-antioxidant-dietary pattern research field began with the studying of the role of ROS in spermatozoa [5], to researching the role of single-antioxidant in male generated ROS infertility [8,9,10], to the more recent analysis involving foods and dietary patterns [11,12]. 

Therefore, the aims of the present review are: (i) to summarize the main sources of ROS in male infertility; (ii) to update the knowledge about the reproductive consequences of excessive ROS concentrations and oxidative stress on the semen quality parameters and Assisted Reproduction Techniques (ART) clinical outcomes, including in vitro fertilization (IVF) and intracytoplasmic sperm injection (ICSI); (iii) to extensively discuss the role of antioxidants individually, and in combination with other antioxidants, and (iv) discuss why diet could be a more useful long term solution for improving oxidative stress and therefore sperm quality results and fertility outcomes.

## 2. Reactive Oxygen Species Related to Male Infertility

ROS are unavoidable by-products created from cellular respiration. They are unstable products, having one or more unpaired electrons, making them highly reactive [13]. ROS are ever-present in the body, acting as signal transducers in the complex biochemical cascade required for sperm maturation. At physiological levels, they play a role in sperm maturation, capacitation, hyperactivation, acrosome reaction, and sperm-oocyte function [14]. An excessive amount of ROS results in oxidative stress [5]; one of the leading causes of male infertility [15,16]. There are many different types of ROS in the human body. This paper will only discuss superoxide (O_2_^−^), hydrogen peroxide (H_2_O_2_) and hydroxyl radical (-OH) as evidence supports the main role they have in human male’s reproduction. ROS O_2_^−^, a highly reactive molecule, is converted to a less damaging form, H_2_O_2_ through an enzyme group called superoxide dismutases (SODs). ROS H_2_O_2_ is converted to -OH, vi multiple reactions, the Fenton reaction being one of them [17]. 

Sperm ROS are generated by both endogenous and exogenous sources. Certain chronic diseases like obesity and diabetes increase the production of endogenous ROS. These diseases highjack the physiological production of ROS and exacerbate its production. Given this fact, chronic diseases are placed in the endogenous sources category in this review. 

### 2.1. Endogenous Sources of ROS

**Figure 1:**
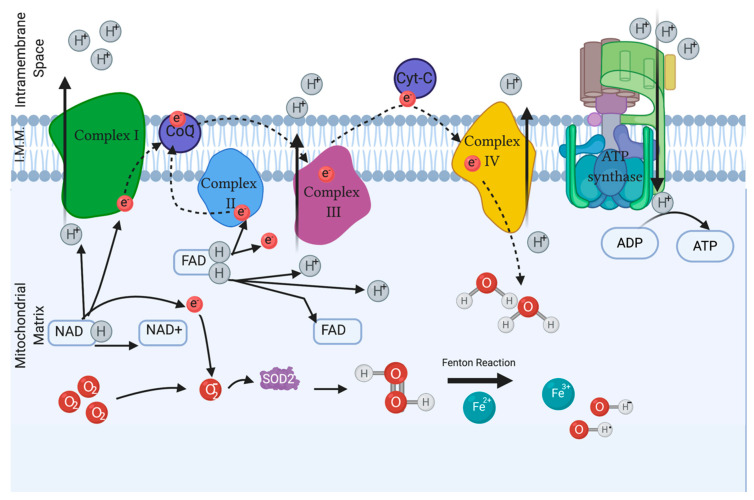
Flowchart of the generation of reactive oxygen species (ROS) during cellular respiration driven by the electron transport chain (ETC) in the mitochondria. Complex I, II, III, and IV constitute the ETC, and ATP synthase completes the oxidative phosphorylation. Fenton reaction constitutes the donation of an electron to transform H_2_O_2_ to two molecules of hydroxyl radicals. Abbreviations: ADP: Adenosine diphosphate. ATP: Adenosine triphosphate. CoQ: Coenzyme Q, ubiquinone. Cyt-C: cytochrome complex. FAD: FADH_2_ reduced form. FADH_2_: Flavin adenine dinucleotide. Fe^2+^: Ferrous ion. Fe^3+^: Ferric ion. H: Hydrogen. I.M.M.: Internal mitochondrial membrane. NAD: NADH reduced form. NADH: Nicotinamide adenine dinucleotide. O_2_: Oxygen. O_2_^−^: superoxide. SOD2: Superoxide dismutase 2 (mitochondrial variety).

Sperm ROS are generated in the mitochondria during aerobic metabolism, via the electron transfer chain (ETC) when the influx of electrons entering and exiting are mismatched [18] and when the natural antioxidant defense is overwhelmed [19]. Complex I and Complex III generate O_2_ as a side-product and release it to the matrix. Complex III also releases this same ROS into the intermembrane space. Alterations in the output or input will unbalance the equilibrium of the gradients and cause a surge of O_2_ production [20]. This process is schematized in Figure 1. The disruption of the ETC results in the accumulation of ROS, causing the fenestration of the outer membrane of the mitochondria, exposing the DNA and promoting the apoptosis of the cell [21]. ROS can easily damage the mitochondrial DNA as it is near the ETC and lacks introns, making it easily prone to oxidation. Both the lack of conventional histone proteins and the limited mitochondrial damage repair capability also aid in making DNA very susceptible to ROS [22]. Furthermore, if the genetic material is damaged, the production of ATP becomes ineffective. 

#### 2.1.1. Age 

Aging, although perfectly physiological, is also associated with an increased production of endogenous ROS and therefore with decreased fertility [23]. It is well documented how fertility diminishes as females age [24,25]. Nevertheless, in males, aging is also strongly related to a general decline in the male reproductive system functionality, sperm quality, and fertility. Some authors suggest that sperm motility, among other parameters, decreases continuously between 22 and 80 years of age [26,27]. As the body ages, the cells do as well; the role ROS play in the aging of spermatic cells is one that involves DNA fragmentation, cell structural damage and therefore a decline in cellular function. There have been several studies that support the aging theory. Different studies have proven the link between advanced male age and DNA damage chromatin integrity, gene mutations, and aneuploidies in sperm [28,29]. Subsequently, researchers have recorded an association between aging, sperm telomere length and embryo quality in in-vitro fertilization (IVF) [30]. More recently, researchers have focused on the relationship of diminishing telomere length and how it culminates in lower motility rate, less sperm vitality, less protamination and more DNA fragmentation [31]. DNA damage is caused by ROS molecules Another finding that relates to aging and infertility is the decrease in testosterone males experience as they age, the consequence being evident in the decrease of antioxidant defense in the Leydig cells [32]. 

#### 2.1.2. Diseases 

Male human infertility may be caused by multiple diseases mediated by ROS. These pathological states can be noncommunicable diseases, obesity, diabetes, and varicocele among many others [23]. 

##### Obesity

The global epidemic of obesity and lowering sperm counts have concurrently become health concerns [33]. In 2016, more than 1.9 billion adults were overweight worldwide, of these, over 650 million were obese [34]. Obesity, defined as a BMI greater than 30 kg/m^2^, affects male fertility in multiple fronts: increased scrotal temperature due to increased scrotal adiposity, hypogonadism, erectile dysfunction and sperm epigenetic changes, among others [35,36]. Obesity is directly linked to a decrease in sperm count and lower testosterone levels compared to healthy non-obese individuals, among other sperm quality parameters and hormonal disturbances [35,37]. Recent evidence suggests that weight has an inverse correlation with sperm count, concentration, motility, vitality, and normal morphology [35,37]. Likewise, BMI has been proven to affect the integrity of spermatozoa’s chromatin, which has a direct influence in the outcome of intrauterine insemination (IUI) [38,39]. The chronic oxidative stress caused by obesity can also affect the testicles and seminal vesicles, causing systemic inflammation [40]. In a recent case-control study, a positive and statistically significant relationship between sperm DNA damage and BMI was reported [40]. Obesity and the increase in ROS production that comes with it damages DNA integrity through multiple pathways [41]. Telomeres are highly susceptible to damage by ROS molecules, affecting the viability of all cell types. This was proven by a 2009 randomized control trial (RCT) that found increased telomere lengths in rectal cells after weight loss in male human individuals [42]. DNA is being constantly repaired; lamentably in overweight subjects the repair pathways are affected, as proven in a non-randomized control trial where nucleotide excision repair mechanism efficiency decreased as BMI increased [43]. 

##### Diabetes

The global prevalence of diabetes has continuously increased over the last decades. In 1980, 108 million people worldwide lived with this disease and in 2015 this number increased to 415 million. Conservative predictions project that by the year 2045, 9.9% of the world population will suffer this affliction [44]. Diabetes mellitus is an umbrella term that includes multiple metabolic disorders that involve insulin resistance and/or deficient insulin secretion, it is characterized by high levels of blood glucose [45]. 

In a recent case-control study involving men with a diagnosis of diabetes mellitus type II, sperm concentration, progressive motility, non-progressive motility, morphology, viability, and DNA fragmentation were found to be worse compared to the control non-diabetic group [46]. A 2002 case-control study found lower sperm motility in insulin-dependent men compared to their healthy counterparts [47]. A recent review broached the question if antidiabetic drugs had the capability of ameliorating diabetic-related male reproductive dysfunction [48]. Some of the research that supports this theory uses animal-based models. In a 2012 study, Akita mice with Type I diabetes were capable of restoring their previously lost fertility after supplementation of insulin, showing histological changes in the structural conformation of the testis and increasing testosterone levels [49]. Diabetes induced murine models supplemented with insulin showed an increase in testosterone bioavailability, and spermatogonial differentiation of primary spermatocytes [50]. The interest in this topic goes back to the previous century; in 1999 a study involving male newts and recombinant human insulin-like growth factor, illustrated the importance of this hormone in the differentiation of spermatogonia [51]. A more recent study involving washed human spermatozoa treated with leptin and insulin showed an increase in sperm motility, as well as ROS and nitric oxide production [52], pointing to the importance of the redox balanced system. Besides the hormonal implications this pathology causes, there are other diabetic-related complications that may cause sexual dysfunction, such as neuropathy and vascular insufficiency [53]. 

##### Cancer

Cancer is one of the leading causes of death worldwide; it was estimated that by the year 2020, 1.8 million Americans would have been diagnosed and 606,520 would have died because of this disease [54]. One of the standard definitions of this disease is the rapid creation of abnormal cells that grow beyond their usual boundaries and can then invade adjoining parts of the body and spread to the organs (the latter process is referred to as metastasizing) [55]. Cellular function is strictly redox regulated; signaling and gene expression are just a couple of processes involved in this balance [56]. An altered redox state has been proven to change the regulation of normal and malignant cell growth [57]. The redox balance is as important in cancer cells as it is in healthy ones; while cells with slightly higher than normal ROS concentrations are related to the etiology of cancer, excessive amounts induce apoptosis. Tumor suppressors regulate the expression of ROS, for example the genome’s guardian (p53) can stimulate or depress ROS levels which in turn can cause apoptosis. ROS levels can also alter the signaling involved in cellular regulation and proliferation. Tumor suppressors are affected by ROS and in turn oxidative stress can also affect tumor suppression, cancer thus involves a cyclical pattern of deleterious feedback [58]. Neoplastic and germ cells share multiple characteristics that are not present in other cells. Some of these processes can perfectly exemplify how an innocuous process for reproduction can turn into a cancerous growth: Immune evasion, meiosis stimulation, migration (similar to metastasis), and global hypomethylation to name some examples [59]. Understanding the overlap of cancer and fertility may help us further out knowledge of both fields. 

##### Varicocele

The most common semi-reversible cause of male infertility is varicocele, defined as the elongation and enlargement of the pampiniform plexus’ veins. Varicocele is identified in 15% of healthy men and 35% of men with primary infertility. An excessive production of ROS is linked to this pathological state [60]. This pathology involves the dilation of the veins in the pampiniform plexus, causing obstruction in the testis tubules and therefore increasing the temperature locally. It is important to note that testicles are privy to a temperature exemption in the body, generally being 2 Cº/3.6 Fº cooler than the rest of the body, in order to function properly. Men with varicocele have higher levels of ROS, increase of DNA fragmentation and DNA methylation changes in spermatic cells [61,62]. These alterations may take place during spermatogenesis and spermiogenesis, as men with varicocele have alterations in Sertoli cells. Spermatozoa from men with varicocele are more susceptible to retain cytoplasmic droplets, which are associated with ROS production and subsequently, DNA damage and therefore defective sperm [63].

### 2.2. Exogenous Sources of ROS

ROS can also be caused by exogenous sources; these may be intrinsic to life as infections and some may be associated with less ideal environmental circumstances like radiation or pollution.

#### 2.2.1. Infections

Infections such as chlamydia, tuberculosis, syphilis, leprosy and mumps orchitis can have significant consequences in male fertility. These diseases both in their acute and chronic presentations can impede a pregnancy from ever occurring [64]. Inflammation and the excess of leukocytes in the seminal plasma (leukocytospermia) also increases the generation of ROS molecules in seminal plasma. The consequences of genitourinary inflammation caused by bacteria or viruses continue to be studied [65]. A chronic inflammation may compromise the testicles causing atrophy or it may involve an obstruction of the epididymis [66]. Any kind of immune response occurring in the testicles can be potentially damaging to sperm cells. Given the fact that approximately 10 to 20% of infertile men have elevated seminal leukocyte parameters [67], infections and its involvement in male fertility continue to be an interesting research topic. Recent publications regarding infections and male fertility include: papillomavirus and its links with asthenozoospermia, increased presence of antisperm antibodies and disruption of the ROS-mediated acrosomal reaction [68], and the relationship of seminal microbiome and fertility in men [69].

#### 2.2.2. Pollution

Environmental pollution is a contributing factor to the decrease in sperm quality [70]. There are many possible contaminants; there are pollutants in the air, the water, the soil and in the foods. Endocrine disruptive chemicals are substances that alter the normal hormone biosynthesis and therefore directly affect reproduction. These chemicals range from pesticides, industrial solvents, to pharmaceutical agents. The age of exposure to these chemicals, the latency of this exposition, and individual genetical predispositions can determine what kind of disruption will occur. Some of the possible side-effects to this kind of exposure are dimorphism, decreasing hormone synthesis, altered DNA methylation in germ cells, etc. [71]. 

There is strong evidence related to the decrease in fecundability and increased spontaneous pregnancy loss in couples exposed to sulfur dioxide, fine particulate matter and/or nitrogen dioxide [72]. Men that were more exposed to these toxics, whether it be occupation-related or living proximity, have an increased frequency of sperm abnormalities. Air pollutants are capable of generating ROS, oxidative stress, and therefore sperm DNA damage, that translates in a decrease in sperm fertilization potential [73]. 

#### 2.2.3. Radiation

The effect radiation has on spermatic cells and their function is documented in multiple articles. In 2014, a systematic review and meta-analysis broached the question of radiation emissions coming from mobile telephones and their effects on sperm quality [70]. Radiation affects tissues in multiple ways, non-thermal interaction, changes to protein conformation and binding properties, and an increase in ROS production [74]. Some of the more statistically significant findings regarding electromagnetic radiation were diminished sperm motility [75,76], reduced sperm viability [75,76,77] and decreased sperm concentration [75]. A more purposeful exposure to radiation occurs during cancer treatment, radiotherapy specifically; higher “dosages” may affect fertility and even cause sterilization [78]. Previous preservation of fertility is of great interest for cancer patients [79,80], and should be discussed with a physician prior to any treatment. It is interesting to mention that radiation may affect sperm cells though mechanisms different than ROS however, here we only mentioned a few examples related to electromagnetic radiation and radiotherapy.

### 2.3. Measuring ROS

ROS are instable substrates, measuring them poses a difficult challenge. Indirect ways of measuring ROS are useful. Thiols, mainly present in cysteine residues, are highly susceptible to oxidation and are used as a reliable indirect marker for oxidative stress [81]. Malondialdehyde (MDA), an index of lipid peroxidation may also be used to measure oxidative stress [82]. A possible way of measuring the effect antioxidants have on oxidative stress is measuring total oxidation status (TOS) or total antioxidant capacity (TAC). As each antioxidant has a different biological composition, induction time of each sample would have to be determined prior to analysis [83].

## 3. Reproductive Consequences of ROS and Oxidative Stress

The previously described pathologies and conditions can alter the levels of ROS molecules from a normal physiological level to a pathological level. The consequences can be seen both in the sperm cells or in the reproductive outcomes. 

### 3.1. Sperm Cells

Semen analysis has typically been used as the gold standard for measuring men’s fertility. The oxidative stress caused by the excessive production of ROS directly affects the quality of the sperm by damaging sperm’s plasma membrane. An increase in sperm OS could significantly impairs sperm function causing a decrease of sperm motility and vitality, among others [4,84,85,86,87]. These impairments could also result in male infertility via mechanisms involving the induction of peroxidative damage to the sperm plasma membrane, DNA damage, and apoptosis [88,89]. There are multiple physiological processes that require the use of low and controlled concentrations of ROS, such as capacitation, acrosome reaction, sperm-oocyte fusion, that can get compromised by an increase of ROS in sperm cells and seminal plasma [90]. Uncontrolled levels of OS therefore can be detrimental not only for fertilization rates, but also in pregnancy and live birth rates [16,91]. It is important to note that when measuring ROS and DNA fragmentation levels in a seminogram the technique used (e.g., TUNEL, Comet, SCSA, SCD, etc.) may cause vastly different results [92]. Washed sperm cell suspensions have the unfortunate consequence of getting rid of the natural antioxidant pathways that could very well protect the sperm from ROS. Oxidative stress cannot be attributed uniquely to the production of sperm cells because ROS molecules are generated throughout multiple different organs in the male reproductive system. For example, polymorphonuclear leukocytes are cells with a major role in the generation of ROS in male infertility [16]. Sperm parameters quality, through a seminogram, only show a superficial state of the spermatic cells [93] and may not be a good predictor of a successful pregnancy [16]. The potential of each sperm cell to function properly may be affected by ROS molecules. Notice that the large and convoluted path of the sperm form the testicles to the Fallopian tube ampulla must be seen as a whole; fractioning this process may cause a failure in fully understating the complex interaction of ROS and antioxidants [16]. One of the challenges of solving male infertility is the lack of mastery of the redox system and how it relates to ideal sperm function [93].

### 3.2. In-Vitro Fertilization (IVF) / Intracytoplasmic Sperm Injection (ICSI) Outcomes

Up to 5% of IVF attempts result in unpredictable failure despite normal sperm parameters [94]. In more than half of these attempts there are also no oocyte anomalies [95]. A possible explanation to this phenomenon could be the higher presence of DNA fragmentation in the spermatic cells; evidence has shown a link between this damaged DNA and lower conception rates in IUI and IVF efforts. In humans, an association between high DNA fragmentation/sperm oxidative stress with higher recurrent spontaneous abortions has also been documented [96]. The main cause of DNA fragmentation in spermatozoa is the excessive amount of ROS molecules and by consequence, oxidative stress [97]. These free radicals can also be generated because of the mandatory in vitro manipulation of the semen during the pre-IVF/ICSI. In fact, several studies found that this manipulation sperm cells are being exposed to high level of supraphysiological ROS causing a significant impact on IVF outcomes [98,99]. A coexistence with DNA fragmentation and low sperm motility, low sperm count and higher amount of spermatozoa abnormal forms also explain the low success in pregnancy and delivery [100]. 

ICSI is also affected for an excessive presence of ROS molecules in seminal plasma and sperm. A probable explanation is the damaged cell development generated by oxidative stress, causing apoptosis and embryo fragmentation. These results suggested that the routine use of sperm DNA testing is therefore well-justified, since it may help improve the efficiency of ART treatments and/or counsel a given couple on the most suitable treatment [101]. A recent systematic review in nonhuman mammals concluded that there exists a negative relationship between sperm oxidative stress and fertilization rates after ICSI treatments [60].

## 4. Antioxidants 

Antioxidants are biological or chemical compounds with the ability to scavenge free radicals and stop the chain reaction that eventually leads to oxidative stress. Infertile men are more likely to have pathological levels of seminal ROS as a result of increased ROS production compared to fertile controls [102]. The relationship between antioxidant use and sperm quality parameters has been vastly studied: there is strong evidence regarding its use in male infertility, particularly in basic semen parameters [9,10,103,104,105,106,107]. Some antioxidants (sodium, potassium, calcium, copper, magnesium, and manganese [108,109]) have insufficient evidence to support their ROS-related infertility role. Inositol is also a promising antioxidant, with in-vitro supplementation studies showing improvement of sperm parameters [22,110]. As the evidence for these antioxidants is mainly from descriptive articles or in-vitro they were not included in this review. Vitamin A is also not discussed as there is not strong enough evidence from clinical studies of its individual antioxidant effect in male human fertility. Further studies of these antioxidants are needed to strengthen their case as potential actors in improving male fertility.

This review will focus on antioxidants with enough evidence of interactions at a spermatic level or in male and couples’ fertility. These male-fertility-related antioxidants are grouped in four categories: physiological enzymatic factors, non-enzymatic factors, micronutrients, and others (Table 1). 

The aforementioned antioxidants will be showed in relation to sperm quality/male fertility by biological nature and their main positive associations or effects, dosage & duration of supplementation, noting the perceived gaps in evidence (summary of the evidence in Table 2).

### 4.1. Physiological Enzymatic Factors

The first group, physiological enzymatic factors, includes: SOD, catalase (CAT) and glutathione peroxidase (GPX).

#### 4.1.1. Superoxide Dismutase (SOD)

**Figure 2:**
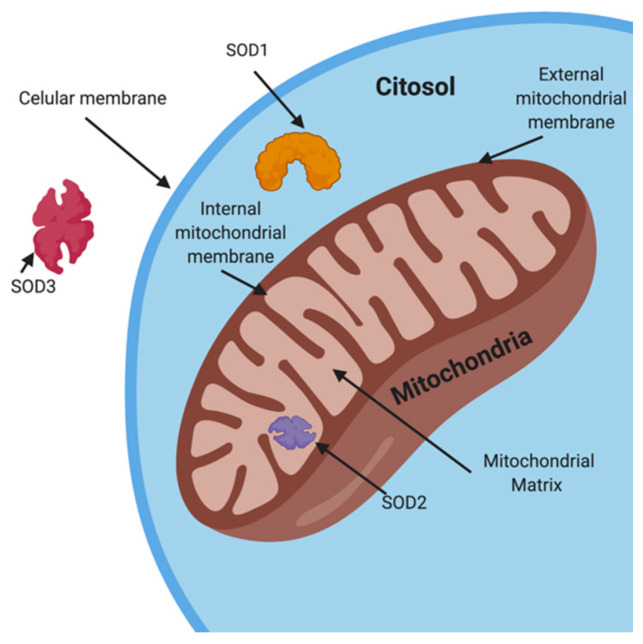
Location of SOD group in relation to cell and mitochondria. SOD1 (Cytosolic SOD or CuZn-SOD). SOD2 (Mitochondrial SOD or Mn-SOD). SOD3 (tetrameric extracellular SOD or EC-SOD).

The SOD group is comprised of three isoenzymes: SOD1/CuZn-SOD, located in the cytosol and responsible for ~75% of the SOD group’s activity, SOD2/Mn-SOD positioned in the mitochondrial matrix, and SOD3 /EC-SOD found in the extracellular space, dissolved in the seminal liquid [111] (Figure 2). Their presence is modulated in response to cellular stress, specifically the presence of O_2_^−^ and lipidic peroxidation [111,112]. The therapeutic usage of SOD enzymes is limited as these antioxidants are highly unstable, have a high immunogenicity and a low circulation half-life. Human-made conjugates of this enzymatic group created with more stability, lower immunogenicity and longer circulating half-life do exist, although their use is limited to animal experimentation so far [113]. Research in animal models has established an improvement in ROS-related chronic diseases such as rheumatoid arthritis [114], osteoarthritis [115], diabetes [116] and diabetic nephropathy [117]. Clinical essays regarding usage of human-altered SOD in male infertility have yet to be carried out. 

#### 4.1.2. Catalase (CAT)

CAT assists the conversion of H_2_O_2_ into molecular oxygen and water. In the male infertility context, CAT has a prostatic origin, being present even in vasectomized individuals. An increased CAT activity is present in normozoospermic individuals compared to their infertile counterparts [118]. CAT’s use as a prolonging agent for sperm survival in artificial insemination in camels has been recorded [119], but its usage in human sperm has yet to be studied. Studies in humans regarding this enzyme range from cell proliferation [120] to pain regulation [121].

A significant alteration in humans is the CAT deficiency (or acatalasemia), an autosomal recessive gene mutation that involves individuals having less than ten percent of CAT enzyme activity [122]. This deficiency was first reported in Japanese patients [123], and subsequently, the disease became known by his name. Swiss [124] and Hungarian [125] families with different mutations in this gene have also been reported. This enzyme’s decline has been associated with multiple chronic diseases such as diabetes mellitus, and hypertension, among others [122]. The relationship between this antioxidant and male human fertility provides a research opportunity for male infertility experts.

#### 4.1.3. Glutathione Peroxidase (GPX)

GPX is a group of enzymes that catalyzes the reduction of hydrogen peroxide to water and oxygen as well as catalyzing the reduction of peroxide radicals to alcohols and oxygen. Research has proven this antioxidant plays an essential role in human fertility. GPX1 levels affect spermatic mRNA, causing poor blastocyst quality, and GPX4 may be used as a chemical marker of sperm maturation. Both GPX1 and GPX4 are linked with a higher sperm recovery after cryopreservation [126]. Cryopreservation, although highly popular in infertility treatments, frequently damages membrane integrity. GPX1 is linked with retaining motility and bioavailability after a cryopreservation-thawing cycle [127]. In animals, specifically boars, GPX5 was found in all the organs of the genital tract, and lack of this antioxidant was associated with embryo-fetal defects, miscarriages and perinatal mortality [128]. 

The selenoprotein phospholipid hydroperoxide glutathione peroxidase (PHGPx) is also part of the family of glutathione peroxidases, serving a role in protecting biomembranes and apoptosis, among others. The latter is an example of the importance of synergy in the human body, as selenium must be present for this specific GPx to work [129]. 

All three enzymes, SOD, CAT, and GPX, work in synergy to reduce free radicals; SOD converts O_2_^−^ to O_2_ or H_2_O_2_, CAT modifies H_2_O_2_ to either O_2_ or H_2_O, and GPX changes H_2_O_2_ to H_2_O [130]. These physiological enzymatic factors are affected by the individual’s health and positively modulating them by an external supplement is not yet possible.

### 4.2. Non-Enzymatic Factors 

Second, antioxidants in the non-enzymatic group are obtained either by endogenous metabolism or by diet. They mainly function by assisting enzymatic factors. This group includes Q-10 coenzyme, carnitine, and lycopene.

#### 4.2.1. Q-10 Coenzyme (CoQ, CoQ_10_)

Q-10 coenzyme, known by its oxidized form ubiquinone or the reduced one ubiquinol, also styled as CoQ_10_ [131], plays a crucial role in protecting the cell membrane from lipidic peroxidation [132]. The very relevant part it takes in the ETC was previously aforementioned in this article. Its antioxidant properties are well studied, and research includes therapeutic interventions in heart [133] and skin [134] conditions. In the male fertility field, a meta-analysis involving CoQ_10_ supplementation was published by Lafuente and collaborators in 2013. This research team concluded that only three RCTs had the quality and relevance to considered [135]. These studies suggest different dosages for CoQ_10_ supplementation during different time frames: 200 mg/day during 24 weeks [136], 300 mg/day for 26 weeks [137], and 200 mg/day for 12 weeks [138]. Safarinejad’s study, 300 mg a day for 26 weeks, showed the most significant improvement in sperm concentration and motility compared with the two others [137]. Pregnancy rate was not increased in any of these three studies. In 2020, a research group redid the meta-analysis broaching the topic of CoQ_10_ supplementation and male fertility as they believed Lafuente’s had used inappropriate statistical measures [139]. Nevertheless, they agreed on the improvement of sperm parameters. The fundamental role CoQ_10_ plays in male fertility and the redox state is proven by the direct correlation between sperm count, ubiquinol and the inverse correlation between hydroperoxide-ubiquinol, respectively [140]. The usage of CoQ10 as a protective entity against oxidative stress and DNA damage has been reported in a 2015 clinical study [141]. Nevertheless, more studies with bigger sample sizes and good methodological designs are needed to further cement this antioxidant’s positive effects.

A recent study [132] aimed to know if a proper dietary intake of CoQ_10_ could show the same improvement as previous studies using supplementation [136,137,138]. Unfortunately, the results concluded that the average dietary intake of CoQ_10_ (mean value of 38.9 mg/day) in men was insufficient to show the expected improvements that supplementation studies showed [132]. The acceptable daily intake of CoQ_10_ is 12 mg/kg/day, with an upper toxic limit of 1200 mg/day; these values are especially relevant as the use of CoQ_10_ has become increasingly popular [142]. This coenzyme may be obtained from the cholesterol metabolic pathway and the diet, mainly from meats, fish, vegetable oils, and nuts. In a lesser proportion, CoQ_10_ is also found in dairy products, vegetables, fruits, and cereals [132].

#### 4.2.2. Carnitines 

Carnitines, also known as l-carnitine or by its active form, l-acetylcarnitine, play an essential role in bioenergy production, acting as a long-chain fatty acid transporter in the mitochondria, protecting cell membranes, and exerting anti-apoptotic actions [143]. They are highly abundant in the epididymis, where they are constantly secreted [144]. A naturally occurring deficiency of this antioxidant is called primary carnitine deficiency. This autosomal recessive disorder is well studied in the Faroe Islands, where several sudden-death cases motivated a nationwide screening. About 55% of the Faroese population, 26,462 individuals, participated in this study, and a prevalence of 1:297 was detected [145].

The positive relationship between carnitines and sperm quality is widely documented. A direct relation between carnitines and sperm motility has been proven in multiple studies [146]. A 2017 study evidenced the positive correlation between seminal l-carnitine and sperm count levels, motility, and morphology [147]. In a case-control study that compared fertile and infertile men, the fertile group had higher seminal carnitine levels (108.43 mg/L), higher sperm counts (66.66 × 10^6^), and higher motility (50.45%) than the infertile group that showed carnitines values of 80.6 mg/L, sperm counts of 52.56 × 10^6^ and motility of 32.31% [148].

Carnitines are obtained 75% from the diet, and 25% is synthesized from lysine and methionine [149]. They are mainly obtained from animal-based foods such as red meat, fish, chicken, and dairy products [150]. The supplemented dosages of l-carnitine range from 2000 mg/day [151] to 3000 mg/day [112] among intervention studies regarding male fertility. 

#### 4.2.3. Lycopene

Lycopene is the primary carotenoid found in the human body, with high concentrations being found in the testes. This lipophilic compound lacks a beta-ionic ring and therefore does not have vitamin A activity like other carotenoid family compounds [152]. This potent antioxidant has antiproliferative, immunomodulatory, and anti-inflammatory effects that promote cell differentiation [153]. In fasting, plasma lycopene is mainly found in line with LDL, HDL, and VLDL concentrations; if there are genetic factors that affect the cholesterol metabolism, the tissue distribution of this antioxidant may be compromised [154]. Evidence suggests that lycopene plays a significant role in the prevention and treatment of chronic diseases, as seen in prostate cancer [155], osteoporosis [156], and atherosclerosis [157].

Regarding male fertility, lycopene supplementation (25 mg once a day) during 12 weeks has proven to improve spermatic count and concentration in a recent RCT, with an average baseline of 49.47 (×10^6^/ejaculate) and 102.45 (×10^6^/ejaculate) postintervention [158]. Another RCT aimed to measure oxidative stress in seminal plasma after 20mg of lycopene supplementation twice a day for 12 weeks, resulting in a decrease in seminal oxidative stress [159]. Another study, without a control group, measured lycopene supplementation (10mg twice a day for three months) and IVF conception success, finding that 7 couples spontaneously conceived during the three-month period before even undergoing IVF [153]. These studies have small samples; larger populations must be studied before drawing conclusions and extrapolating these recommendations to the general population.

Tomatoes are rich in lycopene [158], being the most frequently recommended food source for lycopene increase [155,157,158]. Other red fruits and vegetables such as watermelons, grapefruit, papaya, apricots, and guavas are good sources of this antioxidant [158]. The human body is incapable of producing it as it is only synthesized by plants [152].

### 4.3. Micronutrients

Micronutrients are essential for proper bodily function; an adequate intake is necessary, but excessive amounts may be harmful. This group is subdivided in two categories: vitamins and minerals. Vitamins are organic micronutrients that may be water or fat-soluble and minerals are inorganic micronutrients [160]. 

#### 4.3.1. Vitamins

Vitamins serve an essential role in the human body. The ones most relevant to human male infertility that will be discussed are vitamin C, vitamin E, and vitamin B9 (folic acid). 

##### Vitamin C 

Vitamin C, also known as ascorbic acid, is an electron donor vitamin capable of reducing metals and regenerating vitamin E from its oxidized form. Unable to be synthesized by humans, it needs to be obtained from the diet [161]. The nutritional deficiency of this vitamin, scurvy, possibly the first clinical trial ever made [162], was first described in sailors. 

In sperm cells, vitamin C prevents agglutination and protects against DNA damage caused by ROS molecules [163]. Despite these crucial functions, the verdict of vitamin C supplementation in male fertility is not yet in. Some studies have shown little to no effect in basic semen parameters or DNA fragmentation; only when used in combination with other antioxidants such as vitamin E or selenium, improvements occur [164,165]. Favoring vitamin C as an improver of spermatic quality, a 1990 RCT measured the effects on sperm cells after vitamin C supplementation and evidenced improvement in motility and agglutination with a dosage of 1000 mg a day when compared to the placebo group [166]. Further supporting evidence includes a 2016 RCT involving overweight and obese men supplemented with vitamin C that asserted semen concentration and motility improved [167], and a 2019 prospective cohort demonstrated a positive relationship between vitamin C intake and fertilization rates in couples undergoing ART [168]. 

Proper body reserves of vitamin C are believed to be around 1500 mg, with scurvy appearing in values <300 mg [161]. Vitamin C has a sigmoidal dose-concentration relationship, meaning a small supplementation (<30 mg a day) shows a discreet plasma increase, and a more substantial supplementation (>100 mg a day) does increase vitamin C plasma parameters until hitting a plateau [169]. Good sources of this vitamin include citric fruits like kiwis and mangoes, vegetables such as broccoli, tomatoes, and peppers. Higher-grade evidence research is needed to cement the role this vitamin, standalone, could play in male fertility. 

##### Vitamin E 

Vitamin E is a ubiquitous lipid-soluble antioxidant that protects cell membranes and prevents lipid peroxidation [144]. Although there are multiple tocopherols and tocotrienols in the human body, α-tocopherol is the most active one, therefore commonly regarded as vitamin E [170,171]. Vitamin E cannot be synthesized and must be obtained from the diet [172]. Deficiency is unusual, and rarely due to lack of intake; genetic abnormalities in the alpha-tocopherol transfer protein are the primary cause. Vitamin E deficiency presents itself in the form of neuromuscular abnormalities likely from free radical damage to nerves. Individuals with this avitaminosis need to be supplemented as no optimized dietary regimen is enough to reach optimal levels [170].

This antioxidant serves multiple functions in male fertility, such as testosterone biosynthesis and modulation of telomerase activity [172,173]. In a recent original case-control study involving rats subjected to noise-generated stress and nicotine exposure, the ameliorating effect of vitamin E on sperm viability in subjects under either one of these two stressors was proven [173]. In humans, a prospective study involving healthy individuals showed that vitamin E supplementation of 200 mg/day for 3 months improved lipid peroxidation activity. MDA values decreased, but that did not equate with a reduction of spermatic malformation. Fertilization rates showed improvement after 1 month of vitamin E supplementation but did not continue to improve after more than 1-month intervention [174]. These findings are in accordance with similar studies involving spermatic parameters and vitamin E supplementation [9,10].

Vitamin E is readily available and highly abundant in foods such as nuts, vegetable oils, seafood, cheese, and eggs [172]. In a recent original study, the amount of α-tocopherol in circulating plasma and the dietary vitamin E intake of 641 northern German individuals was assessed. Findings included a reverse trend between a “western” dietary pattern and lower circulating α-tocopherol concentration, suggesting some unhealthy dietary patterns could culminate in an inadequate intake of vitamin E [175]. As long as dietary patterns continue being unknown, the risk Western diets pose to male fertility is a matter for future research.

##### Vitamin B9 (Folic Acid)

Vitamin B9 is known as folate or folic acid (the synthetic form of folate). It is a water-soluble compound [176] essential in DNA metabolism as it is needed in the synthesis of uracil to thymine, protecting against mutations and DNA strand breaks. DNA methylation and gene expression are regulated by this vitamin, preventing abnormal chromosomal replication and mitochondrial DNA deletions [177].

A genetic deficiency of this antioxidant exists; it consists of a defective *MTHFR* gene causing a low concentration of MTHFR enzyme responsible for synthesizing folate or folic acid to l-methylfolate, the biologically active form of vitamin B9. Present in up to 25% of the population, notably Italian, Hispanic, and Asian populations, it causes a diminished synthesis of l-methylfoltate [178]. Other types of deficiencies are caused by chronic alcoholism, malabsorption disorders, higher requirements during pregnancy [176], or using certain medications such as antimalarials, antifolates, and trimethoprim [179]. Pathological states associated with an inadequate intake are macrocytic megaloblastic anemia [176], depression (due to the chemical diathesis in the presence of low dopamine, norepinephrine, and serotonin) [180], dementia, and hyperhomocysteinemia [181]. 

Its usage in improving male fertility has low-grade evidence. A 2002 RCT showed supplementing 5 mg/day of folic acid caused improvement in sperm concentration and normal sperm count, although it was not statistically significant unless used in combination with zinc [182]. On the opposing side, a recent RCT concluded no improvement in semen quality parameters nor an increase in live births with the same supplementation dosage [106]. A 2017 systematic review and meta-analysis on sperm and hormonal parameters in individuals supplemented with folate stated that the only statistically significant improvement found was on sperm concentration [183]. A more recent systematic review and meta-analysis that evaluated the supplementation of vitamin B9 and its effect on sperm parameters found no evidence of improvement in concentration, motility, or morphology [8]. 

It is abundant in foods such as leafy green vegetables (spinach, broccoli, lettuce) and some animal products (liver, milk, eggs) [176]. As neural tube defects grew in prevalence, folic acid supplementation became a public health initiative. Canada and the United States made folic acid fortification mandatory in certain products such as cereals, a notably different approach than Netherlands, where mandatory supplementation is forbidden due to the unforeseen adverse effects on health [184]. High intake of folate in dietary form has no proven adverse effects, contrary to folic acid, where some gastrointestinal events have been reported [176]. 

Although evidence is not enough to support an improvement in spermatic parameters, this vitamin still grants more research in this field as up to 23% of men ages 50-70 have non-optimal folate values, with the cut-off point being <6.8 nmol/L [177], as established by the CDC [185]. Folate serves as a DNA protector only if values are over >36 nmol/L [177]. The effects of low vitamin B9 on DNA sperm integrity are not yet known to our knowledge.

#### 4.3.2. Minerals

Minerals, also known as trace elements, are essential for plant and animal-based life [186]. This section will discuss zinc and selenium as they have a relevant role in human male fertility. 

##### Zinc

Zinc is a micronutrient with reducing properties. It plays a role in signaling, enzymatic activities, regulation of normal growth and sexual maturation, as well as managing mitochondrial oxidative stress [187]. It is estimated that 1/3 of the human population is at risk of being zinc deficient, the most common cause being low intake [188]. Zinc deficiency is linked to ailments such as Alzheimer’s disease, blindness, cancer, digestive pathologies, growth retardation, and inflammation [187]. 

This mineral aids human reproduction in multiple aspects, from a bactericidal effect that protects prostatic fluid from a potential infectious vaginal ambient [189] to maintain the energy system and overall stability until fertilization. Zinc also has an important role in human sperm motility and acrosome reaction [190]. It is widely believed that zinc incorporated into sperm serves to protect against sperm decondensation, aids sperm motility, membrane stabilization, and antioxidant capacity [187]. 

In human male fertility, zinc is involved in multiple aspects, decreasing MDA levels [191], increasing sperm total motility, progressive motility [192], sperm concentration [8], and chromatin integrity [8,193], as well as normal sperm morphology [194]. Low zinc in seminal plasma of infertile men has been vastly reported [93,188,195]. A RCT with asthenzoospermia patients concluded that the supplemented group with zinc sulfate had a higher conception rate (22.5%) compared to placebo (4.2%) [196]. On the other side of evidence, some RCTs show no improvement in sperm quality or ART outcomes after zinc supplementation [106,182]. 

Seminal plasma can benefit from zinc supplementation; unfortunately zinc antioxidant activity does not positively correlate with the dietary intake [197]. As no specialized zinc storage exists in the body, only the daily intake ensures sustained availability [187]. Zinc can be obtained from nuts, legumes, seafood, fortified cereals, and animal products such as meat, yogurt, fish, and milk [189]. Although zinc supplementation has shown improvement in sperm chromatin integrity and increased live birth rates, yet more studies are still needed to certify the improvements zinc could have in male infertility. 

##### Selenium

Selenium is a trace mineral that can target free radicals to suppress testicular toxicity and modulate DNA repair [198]. Selenium, a cofactor of GPX, is also involved in cell-growth, managing cytotoxicity [199], protecting proteins and membranes [200]. 

The selenium pathway may be defective, with mutations in genes such as *SECISBP2*, *SEPSSECS*, and *TRU-TCA1-1*. Selenium deficiency clinically presents as photosensitivity, age-depending hearing loss, and neurodegeneration as the absence of selenoenzymes results in oxidative stress and, consequently, in DNA damage [200]. 

Selenium is positively associated with specific semen parameters such as progressive motility, total motility [199,201,202,203,204], sperm concentration [202,205], total sperm count [202,203,205,206] and normal morphology [202]. Higher live births and a higher pregnancy probability are also associated with higher seminal selenium levels [207]. Nevertheless, supporting selenium’s null effect on sperm parameters, a 2009 RCT showed supplementation of high-selenium yeast in men showed no evidence of improvement in any sperm parameters [208]. 

Selenium seminal plasma concentration is higher within fertile men [203,208], but excessive selenium (exceeding the safe upper threshold of 400 µg per day) also impairs semen quality [205,206], causing semen degradation even in healthy men [208]. The semen selenium range of 50–69 ng/mL gives the maximum benefit in male fertility [206]. In an observational study involving 1136 Chinese men, the average semen selenium amount was found to be 54.32 µg/L [205]. 

A clinical trial in mice showed that nonsteroidal anti-inflammatory drug-related testicular toxicity can be avoided with proper selenium supplementation [198], as these drugs become increasingly frequent, the use of selenium could prove to be an important tool in preventing testicular toxicity.

Humans’ primary source of selenium is obtained through dietary intake. The amount of selenium in certain vegetable foods depends on selenium-rich soil [204]. Fish, garlic, onions and broccoli are some high-selenium foods [209]. 

### 4.4. Others

Finally, antioxidants that do not fulfill the necessary characteristics in the other categories are n-acetyl-cysteine (NAC), melatonin, alpha-lipoic acid (ALA), and w3-fatty acids. 

#### 4.4.1. N-Acetyl-Cysteine (NAC)

NAC, a precursor of GPX that was originally used as a mucolytic drug, can easily penetrate cell membranes [210]. As a derivative of naturally occurring amino acid L-cysteine [211], NAC can directly confront free radicals and stabilize them by donating an electron from its outer layer. 

Multiple studies involving NAC have proven it helps improve male fertility. The use of NAC-incubation on in-vitro human testicular cells reduces the apoptotic rate by 68% compared to controls with no NAC [212]. After NAC supplementation, the TAC of seminal fluid is proven to increase [213,214], as ROS molecules are diminished [214,215,216,217]. Sperm parameters proven to improve from NAC usage are: volume, motility, count, concentration, and normal morphology. Negative processes NAC may aid in diminishing are sperm viscosity, liquefaction time, and DNA fragmentation [8]. 

The great potential NAC may have on male fertility is proven by numerous clinical trials on animals. For example, cadmium toxicity in rats can be greatly reduced when cells are incubated with NAC [218]. Testicular torsion reperfusion generates a vast amount of ROS; the NAC-supplementation group had lower MDA levels in comparison to the non-supplemented group with testicular torsion [210]. Goat testes incubated with malathion, an organophosphate that causes cell death by enhancing ROS production, showed a significant decrease in apoptosis when incubated with NAC [219]. This low-toxicity drug [210] could prove to be an even more substantial aid in counteracting male infertility, as time passes and more studies are conducted, we will discover the full potential NAC may have. 

#### 4.4.2. Melatonin

Melatonin is an amphiphilic hormone, and as such, it can easily pass through cell membranes [220]. Produced by the pineal gland, it helps regulate the sleep-wake cycle [221]; as a pro-sleeping hormone, most of it is secreted during the night [220,221]. Melatonin plays a role in increasing SOD’s, CAT’s and GPX’s activity [221], scavenging ROS formation [222], and even abolishing apoptosis [221].

Fertile men have higher melatonin seminal [223,224] and serum levels [224] than infertile men. This hormone has proven to decrease DNA fragmentation and MDA, and increase sperm viability [221]. Melatonin was shown as well to protect spermatogonia stem cells in-vitro [222]. The disruption of the sleep-wake cycle and its relationship with spermatic parameters was also studied; in a 2020 case-control study men with nigh-shifts or light exposure during the night, showed diminished sperm concentration and motility as well as an increase of abnormal spermatozoa forms [224]. A systematic review and meta-analysis about melatonin and ART concluded melatonin enriched cultures yield higher quality embryos [225]; another interesting experimental study measured microRNAs (miRNAs) in the follicular ambient based on the melatonin profile of female patients, finding miRNAs to be a good non-invasive marker of good quality embryos and melatonin supplementation to yield higher quality oocytes [226]. Incubation of sperm cell with 1 mM of melatonin was also linked with improvement in sperm motility, progression [227] and cell viability [228]. 

Melatonin can be found in multiple plants, but most of them have an insufficient amount to provide to humans [220]; some high-melatonin foods are nuts, red rice, cranberries, and animal products. To obtain effects from diet-based melatonin, these foods must amount to at least 1 mg of this antioxidant and be consumed close to bedtime to help sleep onset [229]. 

There is an interest in the potential melatonin has on male fertility. More research is still needed, as there is still no high-grade evidence regarding oral supplementation of melatonin and sperm quality parameters and DNA fragmentation.

#### 4.4.3. Alpha-Lipoic-Acid (ALA) 

ALA is a potent biological antioxidant, detoxification agent and chelator of redox-active metals [230] that can enter the Krebs cycle, and assist in ATP production [231]. ALA can help create a robust shield on cell’s membranes that can enhance the resistance against free radicals [231]. This natural short-chain fatty acid can also promote the functionality of SOD, CAT, and GPX [232]. Also known as thioctic acid, ALA is able to regenerate vitamin C and E from their respective radical forms and inhibit apoptosis [233].

ALA oral supplementation or cell incubation, is proven to improve sperm quality parameters [234], such as total sperm count [235], concentration [235], motility [235,236,237], viability [236,237] and sperm morphology [232]. In seminal plasma, TAC increases, and MDA decreases after being supplemented with ALA [235]. Regarding ART, ALA can help increase fertilization and implantation rates, increase the quantity of good quality embryos and number of pregnancies, and decrease the occurrence of miscarriages [238]. DNA fragmentation is also reduced both when sperm is incubated with ALA after thawing [237] and with non-frozen sperm [236]. 

ALA can be generated from *de novo* synthesis; it is enough to supply all body requirements. Orally supplied ALA is mostly from supplemental sources as a typical Western diet does not provide a significant amount [230]. No upper limit for ALA has been concretely established in humans [230], although it is recorded that an adult can take a dosage up to 2400 mg/day without experiencing negative side-effects [239]. Clinical trials have shown no side-effects with oral dosages of 600 mg/day [240] to intravenous administration of 1800 mg/day [241]. The interest in generating higher-grade evidence is showed by the existence of a protocol for a future systematic review and meta-analysis that aims to answer the question of efficacy and safety of ALA in male fertility [242].

#### 4.4.4. ω-3 Fatty Acids

Know by multiple names such as omega fatty acids (OFA) [243], or by the less broad term [244] omega-3 polyunsaturated fatty acids (PUFAs) [243], this group has five main constituents: alpha-linolenic-acid, eicosapentaenoic acid, docosahexaenoic acid stearidonic acid and docosapentaenoic acid [245]. Alpha-linoleic acid has the capacity to convert to eicosapentaenoic acid and docosahexaenoic acid, although this ability is limited [245]. 

Higher OFA intake results in increased normal sperm morphology [246,247], volume [248], concentration [246,249], motility [246,249] and total sperm count [246,248]. OFA supplementation has proven to result in higher TAC and lower DNA fragmentation than non-supplemented groups [250]. A 2019 systematic PRISMA review assessed the evidence regarding OFA supplementation and the effects on semen quality markers in infertile men. They concluded that OFA does seem to have a positive effect on sperm quality parameters, although they noted that the available RCT’s are very few and overall lack a substantial number of participants and homogeneous interventions between each RCT. They close with a call for more research and suggested measuring fecundity as a possible outcome [243]. 

Epidemiologic evidence demonstrates that the average American consumes 0.17 g/day of OFA, below the suggested 0.5 g/day [244]. Aquatic organisms are excellent OFA sources; some examples include cod liver, seal and whale blubber, and salmon [245]. Foods with high alpha-linoleic-acid content include flaxseed oil, chia seed, walnut oil, fish oil, and canola oil. As alpha-linoleic-acid conversion to other more bioavailable OFA is limited, an adequate dietary intake of other OFA’s is important [245]. In a 2020 cross-sectional study, sperm quality results of an OFA supplementation were measured in two groups according to the length of the intervention, less than 60 days and more than 60 days. The group with a lengthier supplementation showed significantly improved sperm quality parameters than the <60 days group [248]. The latter suggests a more prolonged OFA supplementation could potentially benefit sperm quality parameters more than a short-term intervention. 

**Table 2:** Main positive associations or effects of single antioxidants related to sperm quality/male fertility by biological nature.

Antioxidant & Doses Relating to Male Fertility	Article	Specie	Level of Evidence	Dose & Duration	Main Conclusions	Gaps in the Evidence
CoQ10 [112] RDD: N/A RSD: 200–300 mg MDD: 12 mg/kg	[135]	Human	Review and Meta-analysis	N/A	CoQ10 supplementation improved sperm motility and concentration.	RCTs with larger sample size, DNA fragmentation consequences, and ART outcomes
	[139]	Human	Review and Meta-analysis	N/A	CoQ10 is positively associated with sperm motility.	
	[136]	Human	RCT	200 mg/day for 24 weeks	CoQ10 supplementation improved sperm motility.	
	[138]	Human	RCT	200 mg/day for 12 weeks	CoQ10 supplementation improved TAC concentrations and decreased MDA levels.	
	[139]	Human	Clinical trial (no control group)	300 mg/day fro 26 weeks	CoQ10 supplementation improved sperm concentration and motility.	
Carnitines [112] RDD: N/A RSD: 3000 mg MDD: 3000 mg	[146]	Human	RCT	25 mg/day for 3 months	Carnitines supplementation improved sperm count and motility.	RCTs with larger sample size, DNA fragmentation consequences, and ART outcomes
	[147]	Human	Observational	N/A	Higher seminal carnitines are positively associated with higher sperm counts, motility and morphology.	
	[148]	Human	Observational	N/A	Higher seminal carnitines are positively associated with higher sperm count and motility.	
Lycopene [251] RDD: Unknown RSD 4-20 mg MDD: Unknown	[153]	Human	Clinical trial (no control group)	10 mg/twice a day for 3 months	Lycopene supplementation increased seminal Omega3.	RCTs with larger sample size, DNA fragmentation consequences, and ART outcomes
	[158]	Human	RCT	25 mg/day for 12 weeks	Lycopene supplementation improved sperm count, concentration, motility; and higher TAC.	
	[159]	Human	RCT	10 mg/twice a day for 12 weeks	Lycopene supplementation decreases seminal oxidative stress.	
Vitamin C [112] RDD: 90 mg RSD:200–1000 mg MDD: 2000 mg	[163]	Human	Review	N/A	Vitamin C is linked to decrease in agglutination and DNA damage parameters.	Higher grade evidence, such as a meta-analysis, RCTs with larger sample size, DNA fragmentation consequences, and ART outcomes
	[166]	Human	RCT	1.0 g/day for 60 days	Vitamin C supplementation improved semen agglutination and increased viability.	
	[167]	Human	RCT	1000 mg of vitamin C were given every other day for 6 months	Vitamin C supplementation improved sperm concentration and motility.	
	[168]	Human	Observational	N/A	Vitamin C intake levels is positively associated with higher fertilization rates	
Vitamin E [112] RDD: 15 mg RSD: 300–600 mg MDD: 1000 mg	[172]	Human and others	Review	N/A	Vitamin E in humans plays a crucial role in the modulation of telomerase activity.	Higher grade evidence, such as a meta-analysis, RCTs with larger sample size, DNA fragmentation consequences, and ART outcomes
	[173]	Albino Wistar Rats	RCT	100 mg/kg /day	Vitamin E supplementation improved sperm motility in nicotine exposed, stress induced rats and rats exposed to both nicotine and stress.	
	[252]	Albino Wistar Rats	RCT	500 mg/kg, 3 times a week for 2 weeks	Histological damage to the testes caused by aluminum was diminished by vitamin E supplementation.	
	[174]	Human	Clinical trial (no control group)	200mg/day for 3 months	Vitamin E supplementation decreased MDA levels and increased fertilization rates.	
	[9]	Human	RCT	600 mg/d for 3 months	Vitamin E supplementation improved sperm cells morphology in-vitro, during the zona binding assay.	
	[10]	Human	RCT	100mg/3 times a day for 6 months or until pregnancy	Vitamin E supplementation decrease MDA levels and improved sperm motility.	
Vitamin B9 [251] RDD: 400 mcg RSD: 400 mcg MDD: 1000 mcg	[182]	Human	RCT	5mg/day for 26 weeks	Vitamin B9 and zinc supplementation improved sperm count.	RCTs with larger sample size, DNA fragmentation consequences, and ART outcomes
	[183]	Human	Systematic Review and Meta-analysis	N/A	Vitamin B9 is positively associated with higher sperm concentration in infertile men.	
	[8]	Human	Systematic Review and Meta-analysis	N/A	Vitamin B9 is positively associated with sperm morphology.	
Zinc [112] RDD: 11 mg RSD: 30–40 mg MDD: 40 mg	[8]	Human	Systematic Review and Meta-analysis	N/A	Zinc supplementation was positively associated with improvements in sperm chromatin integrity index, sperm concentration, motility, membrane integrity, fertilizing capacity, conception, and pregnancy.	Comparative studies determining the best dosage-effect in zinc supplementation.
	[93]	Human	Systematic Review	N/A	Zinc concentration is significantly higher in fertile men.	
	[196]	Human	RCT	250 mg/twice a day for 3 months	Zinc supplementation improved sperm count, motility, fertilizing and reduction in the incidence of antisperm antibodies.	
	[193]	Human	RCT	220 mg/day for 16 weeks	Zinc supplementation improved sperm chromatin integrity.	
	[188]	Human	Systematic Review and Meta-analysis	N/A	Higher mean seminal Zinc levels are found in fertile men. Zinc supplementation is positively associated with semen volume, sperm motility and the percentage of normal sperm morphology.	
	[195]	Human	Review	N/A	Zinc is positively associated with lower ROS production in smokers.	
	[194]	Human	Observational Study	N/A	Higher seminal Zinc is positively associated with sperm count and morphology.	
Selenium [112] RDD: 55 mcg RSDl: 100 mcg MDD: 400 mcg	[199]	Human	RCT	200 μg /day for 3 months	Selenium supplementation improved TAC and sperm motility.	Higher grade evidence, such as a meta-analysis, RCTs with larger sample size, DNA fragmentation consequences, and ART outcomes
	[202]	Human	RCT	200 μg /day for 26 weeks	Selenium supplementation improved sperm concentration, motility, and morphology.	
	[201]	Human	RCT	100 mg/day for 3 months	Selenium supplementation improved sperm count and motility.	
	[206]	Human	Observational	N/A	Higher seminal selenium values are positively associated with sperm count and motility.	
	[204]	Human	Observational	N/A	Higher selenium intake is positively associated with sperm motility.	
	[205]	Human	Observational	N/A	Seminal selenium is positively associated with sperm concentration and total sperm count.	
	[207]	Human	Observational	N/A	Seminal selenium is positively associated with pregnancy and live birth.	
NAC [112] RDD: N/A RSD: 600 mg MDD: N/A	[8]	Human	Systematic Review and Meta-analysis	600 mg/day for 6 months	NAC supplementation improved semen volume, sperm count and concentration, sperm motility, and morphology.	RCTs with larger sample size, DNA fragmentation consequences, and ART outcomes
	[210]	Albino Wistar Rat	RCT	Single dose of 20 mg/kg NAC intravenous	NAC administration improved MDA levels in a postreperfusion testicular injury.	
	[212]	Human	Observational	N/A	NAC incubation reduces the apoptotic rate by 68% compared to controls with no NAC.	
	[213]	Human	RCT	600 mg/day for 3 months	NAC supplementation improved sperm volume, motility, and viscosity, as well as TAC.	
	[214]	Human	RCT	600 mg/day for 3 months	NAC supplementation improved sperm morphology, DNA fragmentation and protamine deficiency. TAC significantly increased and MDA levels decreased under this supplementation.	
	[215]	Human	Observational	N/A	NAC incubation of sperm cells is positively associated with a decrease in ROS production.	
	[216]	Human	RCT	600 mg/day for 3 months	NAC supplementation affects NRF2 expression and therefore decrease in ROS.	
	[217]	Human & Albino Wistar Rat	Systematic Review	N/A	NAC supplementation improved DNA fragmentation indices and ROS production.	
	[219]	Goat (Capra hircus)	Observational	N/A	Sperm NAC incubation resulted in positively associated with a decrease of testicular cell apoptosis.	
Melatonin RDD: Unknown RSD: Unknown MDD: Unknown	[221]	Human	Observational	N/A	Sperm melatonin incubation is positively associated with less DNA damage, and MDA levels; and higher sperm viability.	RCTs with larger sample size, DNA fragmentation consequences, and ART outcomes, and studies without involving alterations in the circadian rhythm.
	[223]	Human	Observational	N/A	Mean seminal plasma melatonin levels are higher in fertile men, with higher sperm motility than infertile individuals.	
	[224]	Human	Observational	N/A	Lower melatonin serum and seminal levels are present in men with oligoasthenoteratozoospermia compared to controls. Melatonin is positively associated with sperm motility.	
	[228]	Human	Observational	N/A	Sperm melatonin incubation is positively associated with higher sperm cell viability.	
	[227]	Human	Observational	N/A	Sperm melatonin incubation is positively associated with sperm motility and less static cells.	
Alpha lipoic acid [232] RDD: Unknow RSD: 600 mg MDD: Unknown	[232]	Human	RCT	600 mg/day for 80 days	ALA improved sperm motility and progressive motility, and less DNA damage.	Higher grade evidence, such as a meta-analysis, RCTs with larger sample size, DNA fragmentation consequences, and ART outcomes
	[234]	Human, rats and boars	Systematic Review	NA	ALA incubation in boars is associated with higher sperm motility, less DNA damage. ALA supplementation in humans is associated with a higher TAC. ALA supplementation in diabetic rats caused increased sperm concentration and motility compared to not supplemented diabetic rats.	
	[235]	Human	RCT	600 mg/day for 12 weeks	ALA supplementation improved sperm count and concentration, higher TAC and lower MDA.	
	[236]	Human	Observational	N/A	Sperm incubation with 0.2 mM of ALA increased sperm viability and decreased DNA damage.	
	[237]	Human	Observational	N/A	Sperm incubation with 0.2 and 0.5 mM of ALA improved the motility, viability and morphology of frozen-thawed specimens.	
Omega3 [251] RDD: Unknown RSD: 200 mg DHA MDD: Unknown	[243]	Human	Systematic Review	N/A	Omega-3 has a positive effect on semen quality markers in semen of infertile men.	Higher grade evidence, such as a meta-analysis, RCTs with larger sample size, DNA fragmentation consequences, and ART outcomes
	[246]	Human	RCT	1.8 g/day for 32 weeks	Omega3 supplements improved sperm concentration, motility and normal morphology.	
	[248]	Human	Observational	N/A	Omega3 (fish oil) supplements are positively associated with higher semen volume, total sperm count, testis size.	
	[249]	Human	Systematic Review and Meta-analysis	NA	Omega3 supplements improved sperm concentration and sperm motility.	
	[250]	Human	RCT	500 mg/ 3 times a day for 10 weeks	Omega3 supplements improved TAC concentrations and reduced DNA fragmentation	

Abbreviations: DNA: Deoxyribonucleic acid. DHA: Docosahexanoic acid. MDA: Malondialdehyde. mcg: micrograms. MDD: Maximum daily Dose. mg: milligrams. N/A: Not applicable. RCT: Randomized Controlled Trial. RDD: Recommended daily dose. ROS: Reactive Oxygen species. RSD: Recommended supplementation dosage. TAC: Total antioxidant capacity. 5. The synergistic effect of concomitant antioxidant supplementation

The use of multiple antioxidants has proven to have a synergic positive effect in improving seminal quality parameters [112]. An excellent example of this potentializing effect is proven with the folic acid-zinc combination. As previously stated, individually, both folic acid and zinc have a low antioxidant effect, but their antioxidant capability increases when used in combination. In 2002, a research group studied the effects the combined supplementation of folic acid and zinc had on fertile and infertile men. This RCT showed a 74% increase in total normal sperm count when comparing preintervention values to postintervention values [182]. This same group published a follow-up RCT in 2006 where folic acid and zinc supplementation consequences in endocrine parameters such as testosterone, inhibin B, and FSH (follicle-stimulating hormone) were studied. This supplementation was not found to cause any endocrine changes, although the increase in sperm concentration was once again found [253]. A 2017 systematic review and meta-analysis proposed evaluating the outcome zinc, and folic acid supplementation had on endocrine and sperm parameters in sub-fertile men [183] Some RCTs described no changes with supplementation in sperm parameters [193] and others found improvements in both oxidative stress [254,255] and sperm parameters [182,253,254,255]. A meta-analysis showed that combined supplementation of folic acid-zinc had a positive effect on sperm concentration and morphology on subfertile men, although a significant weakness was the heterogenic nature of the studies included [183]. Interestingly, a 2020 large RCT further researched the effects the folic acid-zinc duo causes in male fertility parameters and concluded it does not significantly improve semen quality or couple’s live rates [106]. 

Although these authors [106] concluded the use of folic acid and zinc does not improve sperm parameters, the beforehand mentioned studies provide extensive evidence supporting their antioxidant effects, the capacity for improving sperm parameters, and the necessity of studying the synergetic behavior of antioxidants in male fertility.

Antioxidants interact in more than one way; as a case in point, CoQ10 plays an important role in maintaining vitamin C and E in their full active reduced forms [131]. If a key antioxidant is missing it may disrupt other’s normal function; for instance, a clinical trial involving carnitine supplementation improved sperm parameters only in patients with normal GPX levels. This points to the fact that only organisms with proper mitochondrial function may benefit from antioxidant supplementation [256].

Supplement companies take advantage of this synergy, and therefore their products are designed with this in mind. These go from presentations including tomato concentrate, vitamin C, grape seed extract, selenium, vitamin E, B-carotene and others, to capsules made from multiple herbs where each-one is rich in multiple antioxidants [257]. Nevertheless, these companies offer these products without enough scientific evidence to support the benefits or potential damages of these combinations.

In 2019, a comprehensive Cochrane systematic review was published and concluded that exists a relationship between antioxidants supplementation and an increase in clinical pregnancy rates. This review included a total of 61 studies, all of them regarding infertile couples undergoing ART. Unfortunately, only 12 of them reported live birth or clinical pregnancy outcomes. The researchers determined that some antioxidants might increase live births, comparing a 12% increase in the placebo group to a 14-26% increase in the intervention group. Unfortunately, when the studies were further analyzed for risk of bias and removed accordingly, no evidence of increased births was found. The rate of clinical pregnancy may also be improved using antioxidants, comparing an increase of 6% in the non-treatment group with the 12-26% in the intervention group. Adverse effects to the usage of antioxidants were also studied, including gastrointestinal complaints and miscarriage events. The stomach issues had a low incidence in both the placebo and the treatment group. These reports were very different in each study and therefore the evidence was rated as very low. The miscarriages were not found to be more frequent in the treatment group than in the control group. This systematic review concluded that antioxidants might help increase rates of conception and live pregnancies in subfertile males. The evidence is not strong enough to make an asseveration without further studies with a better methodology [7].

## 5. Antioxidant Paradox

The antioxidant paradox is the phenomenon of adverse effects taking place when the equilibrium of the redox system is compromised in favor of a reduced state in the presence of too many antioxidants, causing reductive stress. Our more health-oriented society is prone to acquire over-the-counter antioxidants as they are believed to be “healthy” [258]. Unfortunately, they frequently have unusually high concentrations of purified antioxidants [259], such as vitamin C, vitamin E, and lycopene [258]. Many commonly available food items are already supplemented with antioxidants and vitamins [260]. 

Our poor understanding of antioxidant therapy and each individual’s ideal redox state may cause us to ignore if it is too little or too much of a dosage. Knowing the proper dosage in relation to male fertility of each antioxidant could help us supplement the proper amount needed to improve semen parameters (Table 2). In men, the excessive use of antioxidants can have adverse effects that affect the sperm nucleus integrity, making it less resistant to aggressors. A redox state may affect the epididymal maturation, preventing the formation of disulfide bridges between protamines and therefore making the sperm nucleus less resistant [261,262].

For example, high levels of selenium, an antioxidant with multiple clinical trials proven its beneficial effects in sperm quality parameters, can be detrimental if used excessively. Selenium over ≥80 ng/mL in seminal plasma, higher than the optimal range of 40-70 ng/mL, is associated with asthenozoospermia, and elevated miscarriage rates [258]. 

Furthermore, many antioxidants depend on the synergic action of multiple compounds. If one key component is missing, the others may prove to be toxic, rendering the desired antioxidant effect ineffective [258]. This could explain why antioxidant trials sometimes show beneficial health effects while others show no effects at all or even harmful effects. Pointedly in male fertility, the over-usage of antioxidants may block some of the oxidative pathways needed for a successful conception [263]. 

The relatively easy solution to a nutritional deficit would appear to supplement whatever nutrient is lacking from the diet. However, why is a healthy diet preferred over antioxidants supplementation in terms of sperm quality improvement and fecundability? From 1999 until 2012, approximately 45% of the USA male population has used supplements [106]. Unfortunately, supplements are seen by many people and physicians as a replacement for a healthy diet, which they are not. The bioaccessibility and bioavailability of each antioxidant depend on multiple factors. The absorption process of antioxidant-rich foods, like fruits and vegetables, is complex and not yet fully understood, making the prediction of bioavailability difficult [264]. For example, vitamin E in non-supplemented regular milk is more efficiently absorbed into the human plasma than milk enriched with vitamin E capsules [265].

The regulation of supplements is not as strict as it is in foods or drugs; no prescription is needed to buy them in the USA, neither in most of countries. Supplements vary vastly from each presentation available; the different concentrations of active ingredients can be explained by their different botanical origin, different compositions, and concentrations [258]. Although a cautious use of supplements may improve sperm parameters, unsupervised use can be harmful to patients. A 2020 systematic review compared RCTs using antioxidant supplementation to improve male fertility and found the supplemented doses frequently exceeded the safe upper limit for some nutrients; other troublesome findings were the inclusion of ingredients without reported evidence and ingredients with a sub-optimal dosage [266]. Any trial that proposes measuring the effect of antioxidants on fertility must prove that the intervention/treatment exerts an effect on oxidative stress [159] and is not potentially harmful to the patient.

## 6. Future Directions: Foods and Dietary Patterns in Male Infertility

Antioxidant supplementation as a valuable therapeutic approach for the infertile couple continues being studied. The unknowns are: some doses, length of supplementation, and the characteristics of men that could potentially benefit from this. General recommendations for supplements are not precise enough and antioxidant therapy in fertility needs to be clearly defined. The evidence so far is methodologically less than ideal, mostly by the criteria for patient selection, the determination of which antioxidants to use (either individually or in combination), questionable dosages, relevant variables not being measured (fertilization and pregnancy rates) or insufficient follow-up, small population samples, among other issues [261]. 

Evidence studying the relationship between diet and fertility is rarely regarding foods or dietary patterns and mostly oriented to single nutrients [267]. Humans do not typically consume antioxidants or nutrients in pure form. Foods and the way we consume them, also known as dietary patterns, are more closely related to the reality of nutrient intake. A long-term solution to improving reproductive health in males could be a healthy dietary pattern that allows for continuous intake of antioxidants in their natural form, acting in synergy with other functional components found in the diet. There are few studies regarding this topic. A 2017 systematic review researched dietary patterns, food, nutrients, and their effect on male fertility parameters and fecundability. In terms of food groups, fish, shellfish and seafood, poultry, cereals, vegetables and fruits, and low-fat dairy products have been positively related to sperm quality. However, diets rich in processed meat, soy foods, potatoes, full-fat dairy products, coffee, alcohol and sugar-sweetened beverages and sweets have been inversely associated with the quality of semen in some studies [11]. A 2009 observational study in sub-fertile Dutchmen evaluated dietary intake through a food frequency questionnaire and fertility with a combination of sperm quality (seminogram and DNA fragmentation) and hormonal balance (peripheric blood sample). Two distinct dietary patterns were detected *a-posteriori*, and subsequently, semen parameters were analyzed; accordingly, each one was subsequently grouped in tertiles (low, intermediate, or high category based on their personal score for each respective pattern). The “Health-Conscious Diet,” characterized by a high intake of fish and seafood, fruits, vegetables and whole grains showed lower DNA fragmentation in the highest tertile compared with the lowest tertile of adherence. The “Traditional Dutch” represented a high intake of margarine, mayonnaise and fatty sauces, meat products, potatoes, and whole grains, evidenced an increase in sperm concentration in the highest tertile compared with the lowest tertile of adherence. Each food group was also analyzed individually with DNA fragmentation, sperm volume, sperm concentration, sperm motility, and sperm morphology and the results were similar. Food groups positively associated with higher sperm quality (concentration, motility, and morphology) were fish & seafood, legumes, whole grains, and vegetables. Food groups negatively associated with sperm quality were eggs, mayonnaise & fatty salads, meat products, and non-alcoholic drinks. Lower DNA fragmentation index was associated with consumption of butter, eggs, fish & other seafoods, fruits, legumes, and vegetable oil. A higher DNA fragmentation index was associated with intake of other food groups: margarine, meat products, and sugar & confectionary. The strength of this study lies in the prospective design and the sample size of 161 men, further supporting the positive link between these two patterns and sperm quality [268]. 

A 2018 observational study analyzed semen quality and a-priori male dietary patterns adherence, specifically Dietary Approaches to Stop Hypertension (DASH), Healthy Eating Index (HEI), Alternative Healthy Eating Index (AHEI), and alternate Mediterranean Diet score (AMED). The AHEI diet adherence, based of food and nutrients predictive of chronic disease risk, showed the best results in total sperm count, concentration, and morphology out of the four dietary patterns [269]. The impact that male dietary patterns have on male fertility continues to be studied [270,271,272,273,274]; additional studies in a healthy male population could provide a significant point of comparison with infertile men. 

## 7. Conclusions

The present review is a comprehensive description of ROS’s different sources, the reproductive consequences of excessive ROS and oxidative stress, and the possible treatments of ROS imbalances through antioxidant intake, foods, and dietary patterns to improve male infertility. In summary here we describe that some antioxidants, especially selenium and zinc, ω-3 fatty acids, CoQ10 and carnitines, have been positively related to sperm quality and therefore can help improving male sperm quality and fertility. However, although there has been a steady increase in literature regarding this topic, high-quality, well designed prospective and RCTs including larger patient samples and robust methodological design, considering several confounding variables, are still required to confirm supplementation therapy theoretical beneficial effects on subfertile couples. Moreover, excessive use of antioxidants may be detrimental to the spermatic function and many of the over-the-counter supplements are not scientifically proven to improve fertility. A long term and innocuous solution could be a balanced diet, as it takes advantage of the synergy of multiple antioxidants. More studies in fertile population are needed to determine the optimal dietary characteristics for achieving fertility. Since this is a narrative review and not a systematic review/meta-analysis, the summarized information in the present study should be considered cautiously.

## Figures and Tables

**Table 1:** Antioxidants related to male fertility by biological nature.

4.1 Physiological enzymatic factors		Superoxide Dismutase (SOD) Catalase (CAT) Glutathione Peroxidase (GPX)
4.2 Non-enzymatic factors		Q-10 coenzyme (CoQ10) Carnitines Lycopene
4.3 Micronutrients	4.3.1 Vitamins	Vitamin C Vitamin E Vitamin B9 (Folic Acid)
	4.3.2 Minerals	Zinc Selenium
4.4 Others		N-acetyl-cysteine (NAC) Melatonin Alpha-lipoic acid (ALA) ω-3 fatty acid (Omega3)

## Abbreviations

AHEI	Alternative Healthy Eating Index
ALA	Alpha-lipoic-acid
AMED	Alternate Mediterranean Diet Score
ART	Assisted Reproduction Techniques
CAT	Catalase
CoQ	Coenzyme Q/CoQ_10_
DASH	Dietary Approaches to Stop Hypertension
ETC	Electron Transfer Chain
GPX	Gluthathione Peroxidase
H_2_O_2_	Hydrogen Peroxide
HEI	Healthy Eating Index
ICSI	Intracytoplasmic Spersm Injection
IUI	Intrauterine insemination
IVF	In-vitro fertilization
MDA	Malondialdehyde
miRNA	microRNA
NAC	N-Acetyl-Cysteine
O_2_-	Superoxide
OFA	Omega Fatty Acids
OH	Hydroxyl radical
RCT	Randomized Control Trial
ROS	Reactive Oxygen Species
SOD	Superoxide Dismutases
TAC	Total Antioxidant Capacity
TOS	Total Oxidation Status
